# Pamphlets Received

**Published:** 1854-03

**Authors:** 


### *Pamphlets Received.*

*Report of the Pennsylvania Hospital for the Insane, for the year* 1853. By Thomas S. Kirkbride, M. D., Physician to the Institution.

"At the date of the last Report, there were 215 patients in the Institution: since which 191 have been admitted, and 171 have been discharged or died, leaving 235 under care at the close of the year.

"The total number of patients in the hospital during the year was 406. The highest number at any one time was 248, the lowest was 214, and the average number under treatment during the whole period was 229.

"Of males there was 205, and of females 201."

This Report is a clear and unambitious document.

*Seventeenth Annual Report of the Vermont Asylum for the Insane.*

"During the past year 510 patients have enjoyed the benefits of this institution. There were 351 remained at the commencement of the year, 159 have been admitted, 138 have been discharged, and 372 now remain. Of those discharged, 72 recovered."

*Diseases of the Uterine System as a cause of Physical Degeneracy, with general views on their Prevention and Cure.* By C. D. Griswold, M. D.

Dr. Griswold has here given to the general public, a little monograph on uterine disease. It is a queer subject for popular reading, but it is discussed in a tone of calmness, and entire absence of the usual claptrap so much affected by popular physiological writers. While we doubt whether any good result will obtain from this publication, there is certainly nothing reprehensible about it, and it should not be classed with the "Love, Courtship, and Marriage" productions of the Fowlers, *et id genus omne.*

*A Reply to the Attacks of Dr. Charles Caldwell.* By Lunsford P. Yandell.

Dr. Yandell involved himself in a quarrel with Dr. Caldwell, by some criticisms on his works and teachings. In it Dr. Yandell seems to have conducted himself with dignity, and manifests no vindictive spirit. This reply seems to have been called for by the virulence of the attacks. We trust that the matter, which must have been very disagreeable to all concerned, may rest here.

*Doctors' Commons.* An Ethic Address delivered before the District Medical Society of the County of Burlington, (N. J.,) Jan. 10, 1854. By S. W. Butler, M. D., President of the Society.

A very clever and sensible production, urging the establishment of "Doctors' Commons," or places of rendezvous, in villages and cities, for the resident physicians, where they may meet and maintain a library and museum.

*Gross Literary Fraud Exposed.* Relating to the publication of Worcester's Dictionary in London. Boston: Jenks, Hickling and Swan.

What the fraud is we do not know, not having read the pamphlet.

*An Address to the Public in regard to the affairs of the Medical Department of Hampden Sidney College.* By Several Physicians of the city of Richmond.

Entirely local in its interest. H.

